# Effect of Purified Mushroom Tyrosinase on Melanin Content and Melanogenic Protein Expression

**DOI:** 10.1155/2016/9706214

**Published:** 2016-09-06

**Authors:** Kamal Uddin Zaidi, Sharique A. Ali, Ayesha S. Ali

**Affiliations:** ^1^Biotechnology Pharmacology Laboratory, Centre for Scientific Research & Development, People's University, Bhopal 462037, India; ^2^Department of Zoology & Biotechnology, Saifia College of Science, Bhopal 462001, India

## Abstract

In mammalian melanocytes, melanosome is a highly specialized organelle where melanin is synthesized. Melanin synthesis is controlled by tyrosinase, the vital enzyme in melanogenic pathway. The present investigation is based on an effect of purified mushroom tyrosinase of* Agaricus bisporus* on B16F10 melanocytes for the melanin production via blocking pigment cell machinery. Using B16F10 melanocytes showed that the stimulation of melanogenesis by purified tyrosinase is due to increased tyrosinase absorption. Cellular tyrosinase activity and melanin content in B16F10 melanocytes were increased by purified tyrosinase in a dose-dependent manner. Western blot analysis revealed that cellular tyrosinase levels were enhanced after treatment with purified tyrosinase for 48 hours. Furthermore, tyrosinase induced phosphorylation of cyclic adenosine monophosphate (cAMP) response element-binding protein (CREB) in a dose-dependent manner. The purified tyrosinase-mediated increase of tyrosinase activity was significantly attenuated by H89, LY294002, Ro-32-0432, and PD98059, cAMP-dependent protein kinase inhibitors. The results indicate that purified tyrosinase can be used as contestant for the treatment of vitiligous skin conditions.

## 1. Introduction

Cutaneous pigmentation is a human phenotype determining body complexion and providing protection against ultraviolet ray damage [1, 2]. Melanocyte, the specialized skin cell, is involved in regulating skin color by producing melanin pigment. Loss of melanin in the epidermis can increase the risk of acquiring skin cancers and hypopigmentation like vitiligo [3, 4]. Defect in melanocytes or their functions results in pigmentary disorder leading to enhanced, reduced, or complete loss of skin pigmentation. Individuals suffering from any of hypopigmentary/depigmentary disorders, particularly disfiguring vitiligo, are susceptible to the environmental assaults and cosmetic psychological stress. Thus, upregulating melanocytes activity in terms of growth and pigment synthesis in such condition is important. In the era of modern medicine which is undergoing rapid change where genomic information is being accumulated, the data on vitiligo has not been appropriately archived or systemized for the disease analysis [5, 6]. In melanocytes and melanoma cells, melanogenesis is controlled via a cascade of enzymatic reactions synchronized at the intensity of tyrosinase. The enzyme synthesizes dopaquinone from tyrosine, which is the rate limiting step of melanogenesis [7, 8].

Mushroom tyrosinase has been extensively studied in Eastern Asia like China, Korea, and Japan. While tropical countries such as India, especially in Central India (Madhya Pradesh and Chhattisgarh), are less explored, about 53 edible mushrooms belonging to four orders, 11 families, and 18 genera of basidiomycetes are reported from MP. As stated, it became evident that extracts from* Agaricus bisporus* have been used traditionally as well as medicinally in various ailments such as antitumor, immunomodulatory, hypocholesterolaemic, anti-inflammatory, antimicrobial, and antiviral activities [9]. Despite this, to the best of our knowledge, there are no studies indicating extract of mushroom as melanogenic agent except for the work of Zehtab et al. [10] who reported that mushroom tyrosinase prevented experimental autoimmune vitiligo. Suppression of clinical and histological disease was observed when animal received mushroom tyrosinase but exact mechanism is still unknown so an attempt is made to explore the detailed mechanism of mushroom tyrosinase on B16F10 melanocytes. Furthermore, there are no studies at all on the effect of mushroom tyrosinase on cultured melanocytes to see the efficacy of the mushroom tyrosinase as melanogenic agent.

Thus, the current investigation would provide a benchmark in exploring the diversity of mushroom and the effect of mushroom tyrosinase on cultured melanocytes, for finding out melanogenic agents. The present study was undertaken keeping in view the above lacunae in literature and for the first time B16F10 melanocytes model has been studied in detail to block the signaling pathway and mechanism of induced melanogenesis by lyophilized purified tyrosinase of* Agaricus bisporus*. The findings of the study have provided vital information on these aspects and differentiating results have been obtained. It may be mentioned here that this is the first report of its kind where purified mushroom tyrosinase has been found to cause skin darkening via melanin displacement within the B16F10 melanocytes. The present study on B16F10 melanocytes is the first study on the effect of mushroom tyrosinase especially from* A. bisporus* that can serve as a melanogenic potent to vitiligo.

## 2. Material and Methods

For the present study, the compound, mushroom tyrosinase (lyophilized powder ≥ 1000 units/mg solid), and protein kinase inhibitors, protein kinase A (PKA) inhibitor (H89), protein kinase B (PKB) inhibitor (LY294002), protein kinase C (PKC) inhibitor (Ro-32-0432), and MEK1 inhibitor (PD98059) were purchased from Sigma-Aldrich, St. Louis, Missouri, United States. Goat anti-murine tyrosinase IgG antibody and Alexa Fluor® 594 donkey anti-goat IgG (H+L) (2 mg/mL) were purchased from Life Technologies, North America, United States. Dulbecco's Modified Eagle Medium (AT006A-5L), fetal bovine serum (RM10432-100 mL), Antibiotic Antimycotic Solution 100x (A002-20 mL), Trypsin-EDTA Solution 1x (TCL042-5 ×  100 mL), MTT {[3-(4,5-dimethylthiazol-2-yl)]-2,5-diphenyltetrazolium bromide} (TC191-500MG), 4′,6-diamidino-2-phenylindole (DAPI) (TC229-5MG), phosphate buffered saline (RM7385-1PK), and Trypan blue, Certified (RM263-5G), were purchased from HiMedia Laboratories Pvt. Ltd., Mumbai.

### 2.1. Preparation of Tyrosinase

In the previous study tyrosinase from* Agaricus bisporus *was purified by ammonium sulphate precipitation, dialysis followed by gel filtration chromatography on Sephadex G-100, and DEAE-cellulose ion exchange chromatography [11].

### 2.2. Preparation of Melanocyte Culture

The melanocyte cell line B16F10 was procured from National Center for Cell Science, Pune, and was cultured in Dulbecco's Modified Eagle's Medium (DMEM) containing 10% heat inactivated fetal bovine serum (FBS), 1.5 g/L NaHCO_3_, 2 mM L-glutamine, 10,000 units of penicillin, 10 *μ*g/mL streptomycin, and 25 *μ*g/mL amphotericin B and incubated at 37°C with 5% CO_2_ in a humidified atmosphere. To inhibit the bacterial contamination 2% benzalkonium chloride was kept in incubator. The cells were subcultured in a ratio of 1 : 3 on every third day. For cell expansion and experiments with isolated cells, the B16F10 cells were detached with 1x Trypsin-EDTA (0.25% Trypsin and 0.1% EDTA in Hank's balanced salt solution). After 3-4 passages, the cells were discarded and when necessary the cells preserved in liquid nitrogen were used as fresh culture.

### 2.3. Cell Viability Assay

When 70% confluency of B16F10 melanocytes was attained, trypsinization and seeding were done in 96-well microtitre plates at a density of 10^4^ cells/well in DMEM supplemented with 10% FBS and 10,000 units of penicillin, 10 *μ*g/mL streptomycin, and 25 *μ*g/mL amphotericin B antibiotic solutions. After overnight incubation, media of each well were replaced and the cells were treated with desired stimulants to perform MTT assay [12], to examine any cytotoxic effect of extracted tyrosinase of* Agaricus bisporus *along with standard control tyrosinase (Sigma) in B16F10 cells over the concentration range of 1 to 64 *μ*g/mL at different incubation periods of 24, 48, and 72 hr, respectively. At the completion of incubation stimulant-containing media were discarded and fresh DMEM containing 1 mg/mL of MTT was added to each well and incubated at 37°C for 4 h. The solution was replaced with 0.04 N HCl-isopropyl alcohol solution and further incubated at room temperature for 30 min. Harvested solution was centrifuged at 11337 ×g for 5 min and absorbance of supernatant was measured at 570 nm using microplate ELISA reader.

### 2.4. Tyrosinase Assay of B16F10 Melanocytes

Tyrosinase activity was performed using L-DOPA as the substrate with minor modifications of Sung and Cho [13]. Prior to experiment the B16F10 cells were cultured and 1 × 10^5^ cells/well were seeded in 24-well tissue culture plates. The cells were treated with different concentration range of 1 to 64 *μ*g/mL of extracted tyrosinase of* Agaricus bisporus *along with standard control tyrosinase (Sigma) for 24, 48, and 72 hr. After treatment the cells were washed twice with ice-cold PBS and extracted by sonication and then centrifuged at 11337 ×g for 10 min at 4°C. For tyrosinase activity, 100 *µ*L of extract from each well was added in 96-well microtitre plates and the enzymatic assay was commenced by adding 100 *µ*L L-DOPA solution incubated at 37°C for 1 h. After the incubation, absorbance was measured at 475 nm using microplate in ELISA reader and protein content was estimated [14].

### 2.5. Melanin Assay of B16F10 Melanocytes

Melanin content was determined by the method of Tsuboi et al. [15] with minor modifications; 1 × 10^5^ cells/well were seeded in 24-well tissue culture plates. After overnight incubation, the purified tyrosinase of* Agaricus bisporus *along with standard control tyrosinase (Sigma) was added in different concentration of 1 to 64 *μ*g/mL for 24, 48, and 72 hours. After trypsinization an aliquot was divided into two parts. Five hundred microlitres was used for cell counting and the rest was centrifuged at 1677 ×g for 5 min and lysed with 200 *μ*L of 1 N NaOH stirring at 80°C for 1 h. Melanin content was measured at 405 nm using ELISA reader.

### 2.6. Determining the Mechanistic Action of Purified Tyrosinase via Using Melanin Content and Western Blot Assay

Effects of purified tyrosinase on the different pathways involved in melanogenesis in B16F10 melanocytes were exposed by using the protein kinase inhibitors, protein kinase A (PKA) inhibitor (H89), protein kinase B (PKB) inhibitor (LY294002), protein kinase C (PKC) inhibitor (Ro-32-0432), and MEK1 inhibitor (PD98059). B16F10 melanocytes were pretreated with 3 *µ*M of protein kinase inhibitors at 37°C for 1 hr and then treated with 64 *µ*g/mL of purified tyrosinase. After treatment, the cells were collected and analyzed by using a melanin content assay. For western blot, the cells were homogenized using ice-cold lyses buffer (0.1 M Tris-HCl buffer, 1% Nonidet P-40, 0.01% SDS, 100 *µ*M phenyl methyl sulfonyl fluoride, and 1 *µ*g/mL aprotinin). The homogenates containing 15 *µ*g of protein were separated by SDS-PAGE. After electrophoresis, a gel was transferred to polyvinylidene difluoride (PVDF) membranes (HiMedia) in western blot apparatus (Axygen Scientific Pvt. Ltd., India) at 15 volts for 25 to 30 min. The membranes were washed with 1x TBST pH 7.2 (137 M Sodium Chloride, 20 mM Tris, and 0.1% Tween 20) and blocked with blocking buffer (5% skim milk, 137 M Sodium Chloride, 20 mM Tris, and 0.1% Tween 20) overnight at 4°C. Following blocking, the membranes were washed thrice in 1x TBST for 5 min each at room temperature. Firstly the membranes were washed and then treated with primary antibody buffer of goat anti-murine tyrosinase IgG (1 : 100 dilutions) with gentle agitation at 4°C for 1 h. After incubation with primary antibody, the membranes were washed thrice in 1x TBST for 5 min each at room temperature with gentle shaking, after extensive washing with secondary buffer of donkey anti-goat IgG conjugated with horseradish peroxides (1 : 2000 dilution) and washed with stripping buffer (10% SDS, 0.5 M Tris, 0.8%  *β*-mercaptoethanol, and ultrapure water). The proteins were visualized using Gel documentation analyzer (Bio-Rad Laboratories Pvt. Ltd., India) and densitometry was performed using Quantity One software [16].

### 2.7. Statistical Analysis

The data is presented as mean ± SEM (standard error of the mean), where *n* represents the number of dose concentrations (treated) used for a particular experiment. Comparisons were made between treated and control groups by using Student's *t*-test. All data were analyzed using GraphPad Prism 5 software (UK). *p* < 0.005 indicates statistically significant difference.

## 3. Results and Discussion 

### 3.1. Purified Tyrosinase Stimulates Dendrite Formation in B16F10 Melanocytes

**Figure 1:**
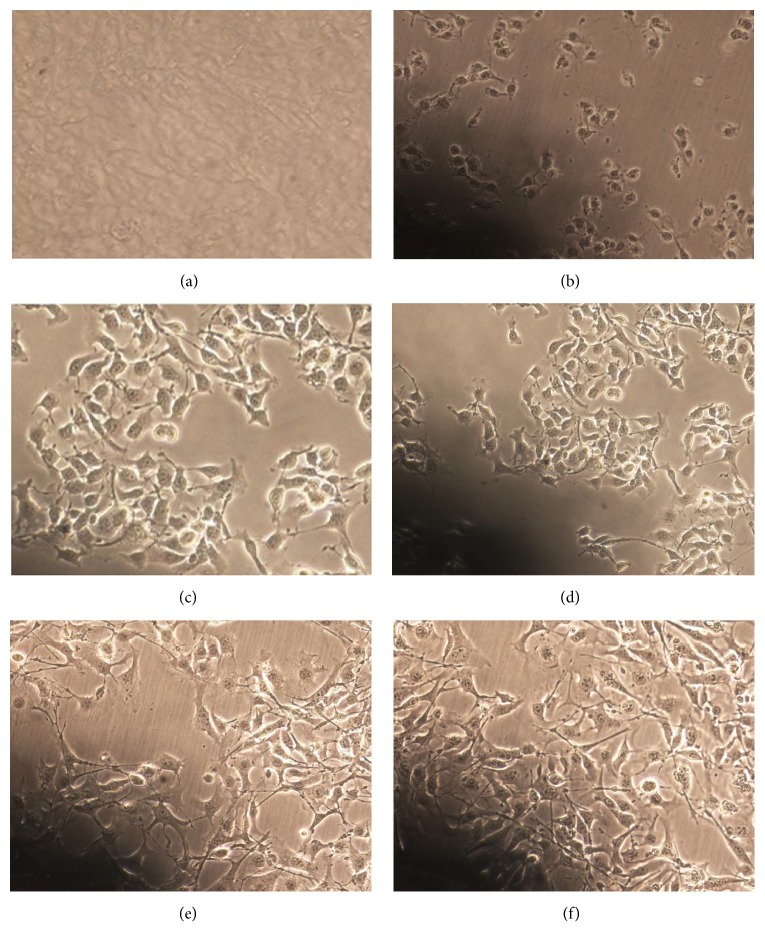
Morphological appearance of B16F10 melanocytes. Melanocytes exhibited no dendrite formation (a). Dendritic network processes in which the pigment granules appeared (b). Increased dendritic process (c, d). Multipolar highly branched dendritic network with dense pigmented granules (e). Increased melanocytes dentricity and proliferation (f). All photographs are under phase contrast microscope equal magnification of 200x.

To assess the functional significance of purified tyrosinase in B16F10 melanocytes, we determined the effects of purified tyrosinase stimulation on B16F10 melanocytes morphology. Prior to treatment B16F10 melanocytes exhibited no dendrite formation (Figure 1(a)). The minimum concentration of 1–4 *µ*g/mL purified tyrosinase of* A. bisporus* caused the morphological change in the B16F10 melanocytes; it was found that the B16F10 melanocytes initially showed dendritic network processes in which the pigment granules appeared on treatment (Figure 1(b)). Increasing the concentrations of purified tyrosinase of* A. bisporus* from 8 to 32 *µ*g/mL, it was observed that there were more pronounced morphological changes, where the melanocytes dendritic process increased further as compared to the untreated B16F10 melanocytes. Thus, there was distinct increase in the dendritic process morphologically, which is exhibited (Figures 1(c) and 1(d)), at the highest concentration of 64 *µ*g/mL of purified tyrosinase of* A. bisporus* under the same culture conditions. It was found that, after the incubation period, the B16F10 melanocytes became multipolar with highly branched dendritic network and also showed dense pigmented granules in the cytoplasm of the treated cells, and clusters of growing cell assembly were also visible (Figure 1(e)). Tyrosinase (Sigma) also increased the melanocytes dendricity and proliferation at concentration of 64 *μ*g/mL (Figure 1(f)). The present data are in fairly good agreement with the suggestion of Mallick et al. [17], who reported the effect of placental total lipid fraction in B16F10 melanoma cells. It was found that B16F10 melanoma cells grew with large dendrites and formed a confluent monolayer and multipolar highly branched dendritic network and also dense pigmented granules appeared in the cytoplasm of the treated cells.

### 3.2. Purified Mushroom Tyrosinase Induced both Cellular Melanin Content and Tyrosinase Activity without Affecting the Cell Viability

**Figure 2:**
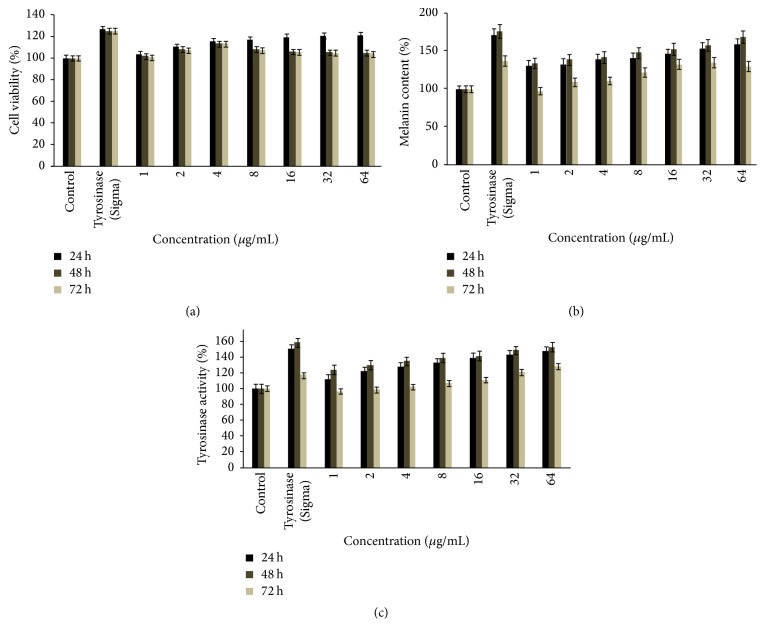
Purified mushroom tyrosinase induced both pigmentation and the cellular content of tyrosinase without affecting cell viability. Cell viability (a). Melanin synthesis in B16F10 melanocytes (b). Tyrosinase activity (c). Data represented as mean ± SE from triplicate experiments (*p* < 0.005).

To examine the cell viability due to the treatment of purified mushroom tyrosinase MTT cytotoxicity assays were performed in B16F10 cells. A wide concentration range of purified mushroom tyrosinase (1–64 *μ*/mL) was preferred. It was observed that cell viability significantly increases in B16F10 culture system at a higher concentration. After 24 h of treatment, all the concentration of purified mushroom tyrosinase (1–64 *μ*g/mL) showed significant cell viability as 103% to 121%, respectively, as compared to control as 100% (0.382 ± 0.5 mg/1 × 10^5^ cells). Maximum cell viability was observed at concentration of 64 *µ*g/mL, 121.2% (0.462 ± 0.5 mg/1 × 10^5^ cells) (*p* < 0.005), whereas tyrosinase (Sigma) possessed maximum proliferation of 126.3% (0.482 ± 0.5 mg/1 × 10^5^ cells) (*p* < 0.005) with respect to control as 100%. Moreover, extending the period of treatment from 24 to 48 h and 72 h and increasing the dose of purified mushroom tyrosinase above 4 *μ*g/mL no further enhancement in cellular proliferation was observed; rather growth stimulation started to decline gradually above this dose reaching 128.9% (0.385 ± 0.5 mg/1 × 10^5^ cells) (*p* < 0.005) as compared to control as 100% (0.371 ± 0.5 mg/1 × 10^5^ cells) value at the considerable higher concentration of 64 *μ*g/mL (Figure 2(a)). These findings are in agreement with those of Mallick et al. [17], who reported the similar effects of placental total lipid fractions on B16F10 melanocytes viability. It had been also reported by Lee et al., 2005 [7], that the serial concentration of glycyrrhizin significantly increased the cell's viability in B16 melanoma cells but significantly decreased when at higher concentration. The present findings are in full agreement with those of Yoon et al. [18] who reported the effects of isopanduratin A on cell viability, percentages of viable melan-cells.

B16F10 melanocytes were exposed to purified mushroom tyrosinase at various concentrations ranging from 1 to 64 *μ*g/mL for 24, 48, and 72 hours. Treatment of B16F10 melanocytes with PMT caused increase in the melanin content in time and concentration-dependent manner. At the concentration of 64 *μ*g/mL, melanin synthesis was stimulated maximally to 159% (*p* < 0.005) with respect to untreated control (100%), while tyrosinase (Sigma) was found to have more potent melanogenic activity in B16F10 cells at 171% (*p* < 0.005) with respect to control. Meanwhile extending the treatment period of purified mushroom tyrosinase from 24 to 48 h at the concentration range of 1–64 *µ*g/mL results in the further enhancement of melanin production, where maximum melanin production was observed at a concentration of 64 *µ*g/mL 169% (*p* < 0.005) and standard control 176% (*p* < 0.005) with respect to untreated control (100%). After the treatment of 72 h, B16F10 cells showed decreased melanin production at all the concentration of purified mushroom tyrosinase 1–64 *μ*g/mL (Figure 2(b)). The present findings suggest that purified tyrosinase induced melanin production in the B16F10 melanocytes significantly; these are well supported by earlier classic work of Raman et al. [19] who reported that aqueous extract of* Angelica sinensis* root, commonly used in the treatment of vitiligo, was very active on mouse melanocytes. Similar data have been reported by Smit et al. [20] where tomato extract was used on the growth and pigmentation of melanocytes. Moreira et al. [21] reported melanogenic activity of hydroalcoholic extracts from the leaves and flowers of* P. venusta* on murine B16F10 melanoma cells. Both extracts increased the melanin content in a concentration-dependent manner on melanoma cells.

To gain insight about the pigment inducing ability of* A. bisporus *tyrosinaseas observed in B16F10 melanocytes, we assessed their effect on the activity of key melanogenic enzyme tyrosinase in the same cellular systems. It was observed that tyrosinase activity of purified mushroom tyrosinase of* A. bisporus* significantly increased at different concentration (1–64 *μ*g/mL) after 24 h of treatment. All the concentration showed increase in cellular tyrosinase ranging from 112% (0.438 ± 0.5 mg/1 × 10^5^ cells) to 148% (0.581 ± 0.5 mg/1 × 10^5^ cells), respectively, as compared to control at 100% (0.438 ± 0.5 mg/1 × 10^5^ cells). Maximum enhancement in tyrosinase activity was observed at concentration of 64 *µ*g/mL, that is, 148% (0.581 ± 0.5 mg/1 × 10^5^ cells) (*p* < 0.005). With extension of treatment period from 24 to 48 h, the tyrosinase activity increased from 124% (0.486 ± 0.5 mg/1 × 10^5^ cells) (1 *µ*g/mL) to maximum tyrosinase activity of 153% (0.601 ± 0.5 mg/1 × 10^5^ cells) at 64 *µ*g/mL (*p* < 0.005) compared to control. After the treatment of 72 h, no further enhancement of cellular tyrosinase was observed (Figure 2(c)). The present finding confirmed that purified tyrosinase increases tyrosinase activity significantly in B16F10 melanocytes is in corroboration with those of Jeon et al. [22] who studied the essential oil from lotus flower extract and its effects on melanogenesis in human melanocytes. It was found that the effective compound induced significant amount of tyrosinase. Similarly, Park et al. [23] had reported that the effect of harmaline and harmalol on B16F10 cells tyrosinase activity occurred in a time-dependent manner.

### 3.3. Mechanistic Action of Purified Tyrosinase Inducing Melanogenesis via Using Melanin Content and Western Blot Assay

**Figure 3:**
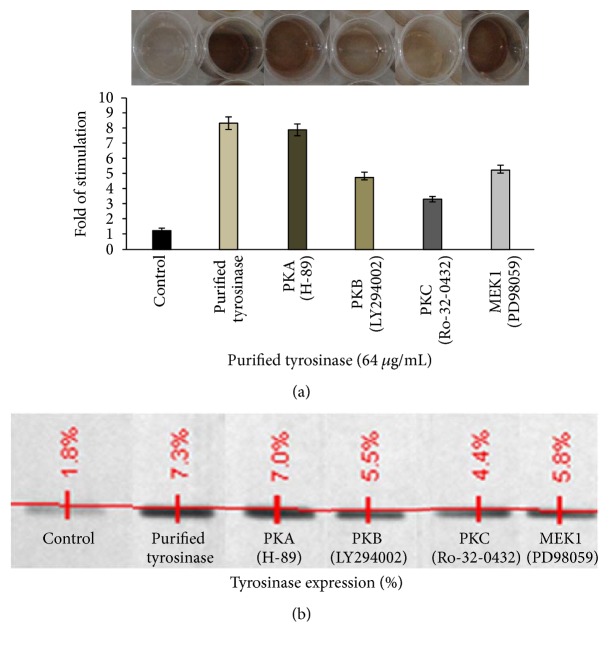
Effects of protein kinase inhibitors on purified tyrosinase induced melanogenesis. Melanin content (a). Tyrosinase expression of treated cells (b). Data represented as mean ± SE from triplicate experiments (*p* < 0.005).

It is well recognized that cAMP accumulation induces activation of PKA, resulting in melanin synthesis. Because activation of CREB is required for cAMP responsiveness of the MITF promoter [24], we investigated the involvement of the cAMP/PKA pathway in purified tyrosinase-regulated melanogenesis; 3 *μ*M of PKA (H-89), PKB (LY294002), PKC (Ro-32-0432), and MEK1 (PD98059) was used to inhibit signaling pathways involved in melanogenesis in B16F10 melanocytes. PKA, PKC, PKB, and MEK1 inhibitors decreased the stimulatory effects of tyrosinase from 8.36-fold value to 7.92-, 4.86-, 3.347-, and 5.31-fold value, respectively. They reduced the percentage of purified tyrosinase-stimulated melanin content from 100% in nonpretreated cells to 94.7%, 58.13%, 40.03%, and 63.51 in PKA, PKC, PKB, and MEK1 inhibitor pretreated cells, respectively (Figures 3(a) and 3(b)). These findings suggest that protein kinase was the major signaling pathway mechanism of purified tyrosinase induced melanogenesis in B16F10 melanocytes. However, the results also show that purified tyrosinase may partially induce melanin synthesis via PKC, PKB, and MAPK signaling pathways.

Protein kinase inhibitors of PKB (LY294002) and MEK1 (PD98069) also slightly decreased the purified tyrosinase increased melanin content and the cellular tyrosinase expressions (Figures 3(a) and 3(b)). These findings were similar to a previous study that demonstrated that cAMP not only initiates PKA activation but also activates other signaling pathways. These pathways include PI3K/AKT and PKB/GSK-3*β* pathways [25].

PKC Ro-32-0432 has been shown to decrease purified tyrosinase-stimulated melanogenesis and expressions of cellular tyrosinase (Figures 3(a) and 3(b)). These results suggest that purified tyrosinase may also enhance melanin synthesis through PKC signaling pathway. The PKC- (protein kinase C-) dependent pathway has emerged as a second intracellular signaling pathway regulating melanogenesis. It was first observed that addition of diacylglycerol, the endogenous activator of PKC, to cultured human melanocytes caused a rapid 3-4-fold increase in total melanin content [26]. Some extracellular signals such as endothelin-1 (ET-1) have been shown to increase melanogenesis by activating PKC. Active PKC activates tyrosinase by directly phosphorylating tyrosinase into melanosome [27].

Since tyrosinase is the key in regulation of melanin production, the study conducted on the effect of purified tyrosinase absorption and its effect has shown mushroom tyrosinase as an effective agent for melanogenesis. The study conducted by Zehtab et al. [10] indicated that the tyrosinase of* A. bisporus* is a key enzyme in melanin synthetic pathway and is expected to prevent autoimmune vitiligo by oral administration. The present study supports that the tyrosinase of* A. bisporus *in B16F10 melanocytes can induce melanin production directly as it was observed by the cells. This might be due to their characteristic similarity to the mammalian tyrosinase;* A. bisporus *has been already reported to be structurally similar to mammalian tyrosinase [28–30]. The physiological role of tyrosinase is related to melanin biosynthesis and has been extracted from different sources such as fungi, fruits, and mammalian melanoma tumors [29, 31]. In addition, tyrosinase is associated with wound healing, with the immune response in plants [32, 33]. In humans, tyrosinase is involved in the pigmentation in melanocytes [34, 35], as a marker in melanoma patients [36] and as a target for the activation of prodrugs [37].

Based on the pharmacological reports presented above, we hereby postulate that purified tyrosinase could have potential of a pigment cell acting drug. In order to confirm the finding, in the present study an attempt has been made to explore the possibility of initiation of the pigment cell machinery by purified tyrosinase, an enzyme from* Agaricus bisporus,* to induce melanin displacement causing skin darkening. It may be mentioned here that this is the first report of its kind where purified tyrosinase has been found to cause skin darkening via melanin displacement within the melanocytes. The mushroom extracts containing the active compound tyrosinase can be used as novel contestant for the treatment of vitiligous skin conditions.
